# Extracellular Vesicles and Their Potential Significance in the Pathogenesis and Treatment of Osteoarthritis

**DOI:** 10.3390/ph14040315

**Published:** 2021-04-01

**Authors:** Anne-Mari Mustonen, Petteri Nieminen

**Affiliations:** 1 Institute of Biomedicine, School of Medicine, Faculty of Health Sciences, University of Eastern Finland, P.O. Box 1627, FI-70211 Kuopio, Finland; petteri.nieminen@uef.fi; 2 Department of Environmental and Biological Sciences, Faculty of Science and Forestry, University of Eastern Finland, P.O. Box 111, FI-80101 Joensuu, Finland

**Keywords:** arthritis, cartilage degradation, exosome, extracellular vesicle, inflammation, mesenchymal stem cell, microvesicles, osteoarthritis

## Abstract

Osteoarthritis (OA) is a chronic joint disease characterized by inflammation, gradual destruction of articular cartilage, joint pain, and functional limitations that eventually lead to disability. Join tissues, including synovium and articular cartilage, release extracellular vesicles (EVs) that have been proposed to sustain joint homeostasis as well as to contribute to OA pathogenesis. EVs transport biologically active molecules, and OA can be characterized by altered EV counts and composition in synovial fluid. Of EV cargo, specific non-coding RNAs could have future potential as diagnostic biomarkers for early OA. EVs may contribute to the propagation of inflammation and cartilage destruction by transporting and enhancing the production of inflammatory mediators and cartilage-degrading proteinases. In addition to inducing OA-related gene expression patterns in synoviocytes and articular chondrocytes, EVs can induce anti-OA effects, including increased extracellular matrix deposition and cartilage protection. Especially mesenchymal stem cell-derived EVs can alleviate intra-articular inflammation and relieve OA pain. In addition, surgically- or chemically-induced cartilage defects have been repaired with EV therapies in animal models. While human clinical trials are still in the future, the potential of actual cures to OA by EV products is very promising.

## 1. Introduction

Osteoarthritis (OA) is the most common joint disease in the elderly and a major cause of pain, disability, and socio-economic burden [1]. It is characterized by synovitis, gradual destruction of the articular cartilage, formation of osteophytes, and remodelling of subchondral bone. The pathogenesis of OA is complex, and age, female gender, obesity, previous joint injury, occupational joint loading, and genetics are among its most important risk factors. Current OA medication and other forms of treatment mostly offer symptomatic relief but cannot repair the cartilage damage that has already occurred, and surgery remains the only presently available effective treatment strategy for late-stage OA. Early diagnosis is of pivotal importance, as substantial joint pathology is usually present at the time of diagnosis. For this reason, biomarkers of early-stage OA are actively sought, and among these, extracellular vesicles (EVs) have recently emerged as potential candidates.

EVs comprise heterogenous populations of nano- and micro-sized membrane-bound particles released by virtually all cell types in both health and disease [2]. They are often classified as exosomes (EXOs), microvesicles, and apoptotic bodies, which differ in size and mode of biogenesis. It would be potentially useful to divide the discussion about the effects of various types of EVs according to this classification. However, the original research articles do not always refer to a specific EV population, as there is difficulty to separate different EV classes from each other with the current methodology. Due to this, it was not feasible to discuss the different EV types separately in this review, and the collective term “EV” is used to describe all these vesicle populations. Still, the majority of the studies were conducted on the EXO class, which can be more or less generalized as the default for the present review.

The composition of EVs reflects but is different from that of the releasing cell [3]. They act as conveyors of RNA, DNA, lipids, and proteins including cartilage-degrading proteinases, and can protect their cargo from degradation [2]. In synovial fluid (SF), EVs also carry hyaluronic acid (HA), a glycosaminoglycan (GAG) that lubricates the surfaces of articular cartilage [4]. The EV cargo secreted from one cell type can be transferred into target cells, where it is able to modulate their gene expression patterns [2]. The number of studies about the roles different EV types play in pathological conditions has recently multiplied, and their function in joint diseases has become a popular research topic. Bioactive molecules that are highly enriched or exclusively associated with OA EVs are actively screened for their potential as diagnostic biomarkers.

Our aim is to review the key literature on the manifestations and possible roles of EVs in the development of OA. We shall assess the sources of EVs in the joint space and the potential impact of these EVs on adjacent tissues, as well as the promising therapeutic interventions that are being developed. We also have a more specific focus, that of not only presenting the reader with the various diagnostic, prognostic, and therapeutic possibilities offered by EVs, but also discussing the potential benefits or redundancy of these novel approaches when compared to existing tools to diagnose and treat OA.

## 2. Literature Search

A PubMed and Web of Science literature search was performed with the following keywords: “osteoarthritis”, “extracellular vesicle”, “microvesicle”, and “exosome”. The search was mostly limited to original research articles and reviews written in English until October 2020. One author (A.-M.M.) screened the titles and abstracts of potential literature and determined their eligibility. The bibliographies of relevant articles were examined for additional references. A total of 64 papers were included in this narrative review. Our intention was not to cite all relevant literature but to choose studies that would help us to gain an up-to-date view of the recent research on the potential effects of EVs on joint health and how these phenomena could be applied to novel diagnostic, prognostic, and therapeutic tools. This remains a timely assessment for a disease that is at the moment treated for symptoms without any curative conservative treatments available.

## 3. EV Signatures in OA and Their Diagnostic Potential

Several cell types present in synovial joints, such as chondrocytes, fibroblast-like synoviocytes (FLSs), and bone, tendon, and ligament cells, are known to secrete EVs [5]. The shedding of EVs can be more active from degraded than from intact cartilage [6]. In SF, EVs are abundant and mainly originate from monocytes, granulocytes, and T-cells, in contrast to plasma EVs that derive from platelets and erythrocytes [7,8,9]. EVs have been proposed to function in intercellular communication and to regulate joint homeostasis by influencing, for instance, cell proliferation, synthesis of extracellular matrix (ECM), and inflammatory processes. Joint tissues can presumably deliver pathogenic signals to each other via EVs but, at present, the dialogue between joint cells and the EVs they secrete remains poorly understood, and the majority of existing literature derives from cell culture studies.

The quantity and composition of EVs in body fluids have been reported to be affected by OA, but these alterations tend to be less pronounced than those observed in the autoimmune-driven rheumatoid arthritis (RA) that is characterized with a higher inflammatory load than OA [10]. In fact, EV levels are often higher in RA SF compared to OA SF [11,12]. Compared to controls, the expression of EXOs was higher in SF, but not in plasma, from patients with early and late-stage OA [13]. EXO counts in SF were also noted to increase from early to severe knee OA without changes in the average particle size [14]. Some other studies found no differences in EV size or concentration between the SF of OA and non-OA-patients [15,16,17]. Neither was there variation in the SF EV counts from patients with primary or post-traumatic OA [4]. According to Boilard et al. [18], platelet-derived (CD41^+^) EVs were not present in the SF of OA patients in contrast to RA or some other forms of inflammatory arthritis. In serum, OA patients can have elevated levels of CD3^+^ CD4^+^ EVs from T-cells compared to healthy controls [19]. Moreover, increased levels of annexin V^+^ EVs and annexin V^+^ CD45^−^ CD61^+^ platelet-derived EVs were documented in the plasma of OA patients compared to healthy controls [12].

EVs could have potential as diagnostic biomarkers of OA, if their cargo reflected the pathological condition of the parental cell. Theoretically, EV contents could vary between different joint diseases and with disease progression, as well as contribute to pathological processes. Moreover, EV cargo may be utilized to screen individuals predisposed to OA before any initiation of cartilage damage. If EVs were to be used to supplement current diagnostic tools, early-stage OA with its EV manifestations would be the obvious target. In contrast, advanced OA is easily diagnosed with conventional radiography and to supplement that with EV tools would be mostly redundant. Non-coding RNAs (microRNA [miRNA] and long non-coding RNA [lncRNA]) are currently among the most intensively studied molecules in EV research. There are several miRNA candidates with potential to inhibit OA progression as well as those that may promote OA by regulating chondrocyte proliferation and apoptosis, ECM metabolism, and inflammatory processes [20].

EV–miRNA can be highly related to OA, and among specific miRNAs upregulated in the SF of OA patients are, for instance, miR-16-2-3p, miR-155-3p, miR-210-5p, and miR-504-3p, and those downregulated include miR-26a-5p, miR-146a-5p, and miR-6878-3p among others [16]. The SF EVs show clear gender-related differences in their OA-specific miRNA profiles. In women, OA-related miRNAs were estrogen responsive and targeted Toll-like receptor signalling pathways. In both genders, downregulated miRNAs were associated with glycan degradation, cell adhesion molecules, and mucin type O-glycan biosynthesis, whereas those that were upregulated were related to thyroid hormone synthesis, biotin metabolism, and amphetamine addiction signalling. In OA chondrocyte-derived EXOs, miR-372-3p is upregulated [21], while miR-95-5p is downregulated [22]. Glycogen synthase kinase-3*β* regulates the OA-specific exosomal expression of miR-372-3p and has potential to influence chondrocyte proliferation and apoptosis [21]. On the other hand, miR-95-5p may regulate chondrogenesis and cartilage homeostasis by targeting histone deacetylase 2/8 [22]. miR-200c is an example of a miRNA species that has been proposed as a potential biomarker for the development of OA [15]. Regarding plasma EXOs, OA was characterized with lower expression of miR-193b-3p compared to control subjects [23]. This miRNA can regulate mesenchymal stem cell (MSC) chondrogenesis and primary chondrocyte metabolism by targeting histone deacetylase 3.

Similar to miRNAs, lncRNAs are not only potential biomarkers of OA, but they could also participate in disease processes. Several different lncRNA molecules may inhibit (e.g., FOXD2-AS1, HULC) or promote (e.g., DANCR, HOTAIR) the progress of OA [20]. Different lncRNAs can affect the survival and proliferation of chondrocytes, ECM degradation, and inflammatory response by targeting various miRNAs and, for instance, matrix metalloproteinase (MMP)-13, Toll-like receptor 4, Janus kinase 1–2, signal transducer and activator of transcription 3, and *β*-catenin. Among lncRNAs, exosomal PCGEM1 was suggested to be an indicator of different stages of OA [13]. Its expression increased in SF EXOs from controls via early OA to late-stage OA and correlated with the WOMAC OA index. EXOs from FLSs enhanced the cellular proliferation and migration of chondrocytes as well as inhibited ECM degradation indicated by the increased expression of COL2A1 (collagen 2A1) and ACAN (aggrecan), the reduced expression of MMP-13 and aggrecanase ADAMTS-5, and the elevated concentrations of sulfated GAGs (sGAGs) [24]. These therapeutic effects were associated with exosomal lncRNA H19 that targets the miR-106b-5p/tissue inhibitor of metalloproteinases 2 axis.

Regarding protein composition, EVs are known to transport cytokines, chemokines [14], and proteinases [25] that could play crucial roles in the amplification of inflammation and in matrix remodelling, phenomena that are typical of OA. The protein profiles in serum EXOs were altered in OA patients compared to controls, and OA was characterized with increased levels of cathepsin F and reduced levels of immunoglobulin alpha-2 chain C region [26]. Articular cartilage vesicles (ACVs) from OA patients had lower levels of, for instance, matrix proteoglycans (PGs) and COL2, and elevated levels of immunoglobulins, complement components, fibrinogen, apolipoproteins, transforming growth factor *β* (TGF-*β*)-induced protein *β*ig-H3, integrin-binding protein DEL-1, vitronectin, and serine protease HTRA1 compared to normal ACVs [27]. The observed proteome could reflect ACV functions, for instance, in mineralization, inflammation, ECM catabolism, cell adhesion, and immunomodulation. Other EV proteins relevant to OA include the channel protein connexin43 that is associated with senescence [28]. In respect to OA progression, SF EXOs were documented to contain higher levels of numerous cytokines and chemokines in patients with severe knee OA (Kellgren–Lawrence scale 3–4) compared to those with early OA (scale 1–2) [14]. These EXOs are likely to promote joint degradation by recruiting inflammatory cells and by inhibiting cartilage proliferation and repair.

Data on the EV lipid composition are scarce regarding OA, yet this issue could be of importance as lipids are not only structural constituents of the EV membrane but also potential cargo that may contribute to inflammatory processes and pain in diseased joints [29,30]. EVs from leukocytes were documented to carry the pro-inflammatory polyunsaturated fatty acid (PUFA) arachidonic acid to OA synovial fibroblasts to be subsequently converted into prostaglandin E_2_ (PGE_2_) via upregulation of cyclooxygenase-2 (COX-2) and microsomal PGE synthase-1 (mPGES-1) [29]. PGE_2_ is known as a key player in joint diseases, but it has complex effects on inflammation, FLS proliferation, and cartilage degradation that cannot be defined as purely detrimental [31]. In addition to fatty acids, EVs have the ability to transport PUFA-derived eicosanoids, enzymes involved in eicosanoid synthesis, and precursors for specialized pro-resolving mediators (SPMs), such as resolvins [30]. Transcellular synthesis of bioactive oxylipins may exert potentially deleterious or beneficial influence on OA tissues. Determining the significance of lipid and fatty acid composition of EV membranes is still at its earliest stages of research regarding joint diseases but should become a focus of future studies. It should also be recalled that ultracentrifugation can result in the co-isolation of lipoproteins with EVs potentially confounding the lipid and protein profiling of the EV fraction [32].

To sum up, OA can be characterized with altered EV quantity and composition in SF and circulation. However, the diagnosis of early OA still lacks specific and effective molecular markers in body fluids, especially circulatory, that could be sampled in a relatively non-invasive manner. Many studies have been conducted on SF that is unlikely to be routinely used for diagnostic purposes. Compared to SF, the sampling of blood and urine would be much more practical and, thus, their EV populations should be screened in more detail, also for gender-specific diagnostic markers. There are several, potentially useful miRNA candidates for diagnosing OA. It must be acknowledged that several of the studies referenced above have focused on small-sized EVs, i.e., EXOs, but biofluids also contain other subpopulations of EVs. In addition, some of the obtained results do not overlap. The diagnostic feasibility and value of EVs in OA remain to be determined in the future.

## 4. Functions of EVs in Osteoarthritic Joints

EVs have been shown to enter chondrocytes, FLSs, and macrophages [6,9,16,24,33]. These cell types have an intimate relationship in synovial joints and their interactions via EVs within SF probably play a vital role in OA pathogenesis [15,34]. The penetration of EVs into cells appears not to be passive diffusion, and it can be stimulated by inflammatory conditions [9]. Interleukin (IL)-1*β* and tumor necrosis factor *α* (TNF-*α*) are mediators of joint inflammation and often used in cell culture experiments to mimic arthritic environment. The presence of HA matrix around OA FLSs can also facilitate the internalization of EVs suggesting that the HA-coat could function as a natural sponge for EVs [33]. The entry of EVs into cartilage has been demonstrated to occur also in vivo in arthritic mice [9].

EVs have been observed to influence FLSs and cartilage in several ways, many of which are detrimental to joint health. EVs from immune cells induced the synthesis of several cartilage-degrading proteinases (MMP-1, MMP-3, MMP-9, MMP-13) and inflammatory mediators (IL-6, IL-8, monocyte chemoattractant protein [MCP]-1, MCP-2) in synovial fibroblasts from OA patients [35]. Increased release of inflammatory factors (IL-6, IL-8, MCP-1, soluble intracellular adhesion molecule-1, vascular endothelial growth factor, chemokine (C-C motif) ligand [CCL] 5) could be observed from FLSs of arthritic patients incubated with autologous SF EVs [8]. Platelet-derived EVs from RA SF also induced the release of IL-6 and IL-8 from FLSs [18].

Articular cartilage contains ACVs that can serve as foci of pathologic calcium crystal deposition [36]. When normal articular chondrocytes were treated with EXOs from normal synovial fibroblasts stimulated with IL-1*β*, they upregulated TNF-α and genes of cartilage-degrading proteinases (MMP-13, ADAMTS-5) and downregulated those encoding ECM components (COL2A1, ACAN) [37]. Increased PG release was also observed from cartilage explants. The treatment of healthy articular chondrocytes with EVs from OA SF resulted in decreased cell survival and anabolic gene expression (COL2, ACAN) together with increased expression of TNF-*α* and elevated activities of MMP-2 and MMP-9 [16]. Song et al. [21] documented decreased cell proliferation and increased apoptosis of normal chondrocytes after exposure to OA EXOs. EV production was elevated from senescent OA chondrocytes, and these EVs were able to transmit senescent characteristics to neighboring cells and to decrease their PG production [17]. Furthermore, SF EVs from aged mice resulted in OA-like pathology and pain when injected into the articular space of young mice.

In addition to FLSs and chondrocytes, EVs have been shown to activate immune cells. SF EXOs from OA patients increased the release of cytokines (IL-1*β*, IL-16), chemokines (CCL15, CCL20, chemokine (C-X-C motif) ligand 1), and metalloproteinases (MMP-7, MMP-12) by M1 macrophages, which play a key role in the pathogenesis of OA [38]. EXO-like EVs from IL-1*β*-treated (OA) chondrocytes stimulated inflammasome activation and the production of mature IL-1*β* in macrophages [6]. Moreover, intra-articular injections of these EVs aggravated synovitis and cartilage erosion in mice with surgically-induced OA. SF EXOs from patients with severe knee OA promoted chemotaxis of lymphocytes and inhibited the proliferation of chondrocytes [14].

While the above-discussed list of deleterious influence of EVs on joint components is a long one, they can also induce beneficial effects. Neutrophil-derived EVs enriched with the pro-resolving protein annexin A1 activated anabolic gene expression (COL2A1, transcription factor SOX9) in chondrocytes, resulting in ECM deposition and cartilage protection via TGF-*β* induction [9]. The release of IL-8 and PGE_2_ and chondrocyte apoptosis were reduced. The administration of EVs to murine arthritis models decreased the loss of sGAGs from cartilage. Intra-articular EXOs from primary chondrocytes cultured in normal conditions were able to prevent the development of surgically-induced OA in a mouse model [39]. They also polarized the macrophage response towards an M2 (anti-inflammatory) phenotype in cartilage and synovium. In vitro, these EXOs delayed IL-1*β*-induced chondrocyte degeneration by increasing cell viability and the expression of COL2 and ACAN and by reducing the expression of MMP-13 and ADAMTS-5. They also restored mitochondrial dysfunction. Moreover, EXOs with SOX9 mRNA from M2 macrophages stimulated the expression of COL2A1 and ACAN in primary chondrocytes, as well as increased the production of sGAGs and COL2 [40]. EXOs from platelet-rich plasma (PRP) increased the proliferation and migration of primary chondrocytes treated with IL-1*β*, and decreased apoptosis and the release of TNF-*α* [41]. In vivo, these EXOs prevented the development of surgically-induced OA in rabbits. Beneficial effects of EVs are discussed in more detail in Section 5.2 and Section 5.3 on the therapeutic potential of MSC-derived EVs.

**Figure 1:**
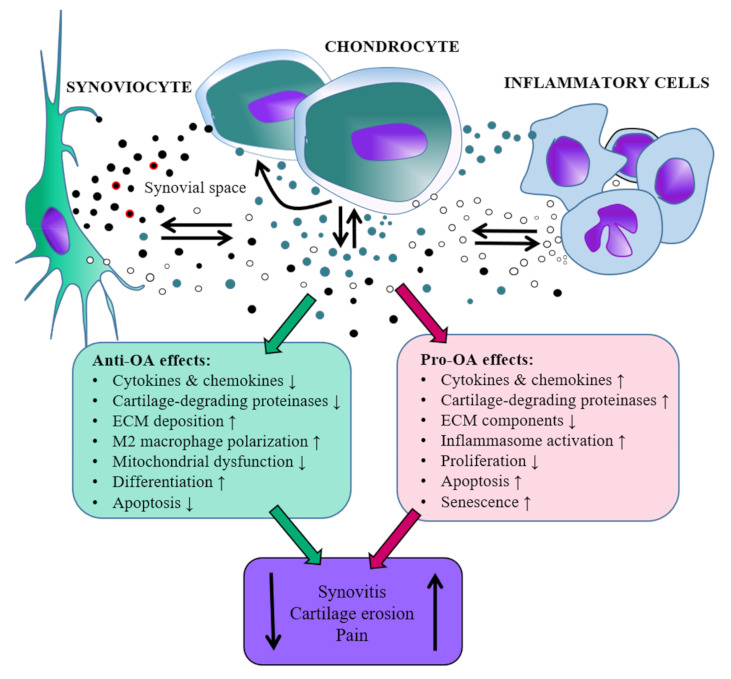
Schematic representation of the roles that extracellular vesicles released by synovial joint cells could hypothetically play during the development of osteoarthritis (OA) based on the literature in the present review. Red coating around EVs depicts hyaluronan. ECM = extracellular matrix, ↓ = decrease, ↑ = increase, note that the scaling of the relative sizes of the image components is not realistic.

In summary, EVs transport biologically active molecules between joint tissues, and they may contribute to the propagation of inflammation and cartilage destruction in inflamed joints by enhancing the production of inflammatory mediators and degrading proteinases. It appears that EVs can contain not only pro-OA but also anti-OA (i.e., therapeutic) properties opening up possibilities of disease amelioration (Figure 1). However, it remains currently unknown how to increase the proportion of the beneficial EVs in the joint space.

## 5. Therapeutic Applications of EVs

### 5.1. Current Treatment of OA

The current state of OA treatment focuses on the alleviation of symptoms, i.e., pain, as a result of joint inflammation, and the maintenance of joint function, the patient’s physical activity, and independence until aggravating symptoms lead to joint replacement therapy. As an example, the treatment options for the symptomatic OA of the knee, one of the most commonly affected joints [42], are listed below based on the evidence-based guidelines by the American Academy of Orthopaedic Surgeons [43]. The recommended treatments include self-management, such as strengthening, low-impact exercise, neuromuscular education, physical activity, and weight loss in case the patient’s body mass index is ≥25 kg/m^2^. Symptomatic pain is strongly recommended to be treated by reducing inflammation with non-steroidal anti-inflammatory drugs (NSAIDs, oral and/or topical) or through the opiate systems with, e.g., tramadol, while the evidence for the use of acetaminophen/paracetamol is inconclusive. In addition, acupuncture, glucosamine, chondroitin, or HA cannot be recommended, and the evidence regarding intra-articular corticosteroids is inconclusive. Obviously, the choice of therapeutic measures is a debated issue and many of the above-mentioned treatment options that are recommended against are still commonly in use [44]. Conservative or minimally invasive therapies are still not totally risk-free, for instance, with the well-known gastrointestinal side-effects of NSAIDs and the risk of accelerated cartilage damage with corticosteroids [44,45].

**Figure 2:**
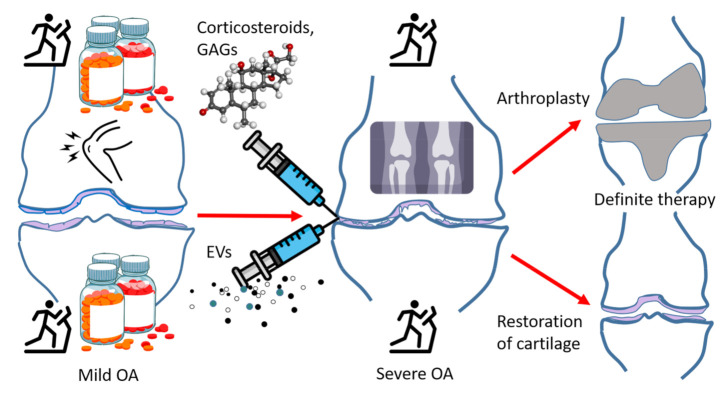
A general scheme depicting the progression of osteoarthritis (OA) and its therapies. Upper half of the image: currently available therapies that eventually lead to knee replacement surgery. Lower half of the image: potential extracellular vesicle (EV)-based therapies and the possible outcome of cartilage regeneration, GAGs = glycosaminoglycans, open source images provided by: https://www.stockio.com/free-icon/healthy-icons-syringe; https://www.flaticon.com/free-icon/x-ray_2286250; https://www.pinclipart.com/downpngs/iTxJwJo_red-pill-bottle-clipart-png-download/; https://upload.wikimedia.org/wikipedia/commons/thumb/0/0a/Methylprednisolone.png/215px-Methylprednisolone.png; https://www.flaticon.com/free-icon/stick-man-running-on-a-treadmill_31417?k=1608198863143; https://uxwing.com/knee-pain-icon/ (all image sites accessed on 29 March 2021).

Surgery may become indicated after previous conservative treatment including the above-mentioned options, definitive knee OA, and persistent pain [46]. While joint replacement yields significant alleviation of pain, patients tend to show reduced activity, and stiffness and unresolved pain are not uncommon. Thus, existing therapies are at first symptomatic, their main goal is to maintain joint functionality, slow down the progression of the disease, and eventually replace the damaged joint with a prosthesis. The failure risk of a knee implant is approximately 1% per year [47]. The medical use of EVs has potential to affect all these treatment options in the not-too-distant future. EVs may: (i) offer pain relief, (ii) decelerate cartilage destruction, and ultimately also (iii) enable the replacement of damaged cartilage with new tissue instead of an artificial knee prosthesis (Figure 2). Here, in addition to discussing the potential tools provided by EVs, we aim to compare their utility to existing therapies and to preliminarily assess if they are not only theoretically feasible, but if they would provide additional benefits compared to existing diagnostic and therapeutic tools in various stages of joint disease.

### 5.2. Attenuation of Pain, Inflammation, and Degeneration by EVs

Pain relief in OA and other joint diseases (principally RA) consists of the use of NSAIDs and physiotherapy, preferably in combination. At the moment, EVs are being investigated for this purpose in different models of OA. In the lumbar facet joint OA model, bone marrow MSC (BMSC)-derived EXOs delivered intravenously to mice were able to relieve chronic pain at 2–5 weeks post-surgery [48]. This promising result was suggested to be caused by the attenuation of aberrant nerve invasion and angiogenesis in subchondral bone accompanied by reduced articular cartilage degeneration and subchondral bone erosion. Similarly, He et al. [49] used the rat knee OA model to assess pain among other therapeutic goals after treatment with intra-articularly administered BMSC EXOs. They noticed significant alleviation of pain responses 2–6 weeks after the model establishment. In both studies, reductions in the protein levels of calcitonin gene-related peptide were observed and suggested to be among the mechanisms for the beneficial effects.

One of the early targets that could attenuate or prevent cartilage destruction would be to block the inflammatory cascade that eventually causes the symptoms of OA. Hitherto, most of the work done to study this issue has been conducted in vitro. Although a definite cure may still be a long way off, promising results are emerging. In fact, treatment with BMSC EXOs has been shown to dampen the inflammatory upregulation of MMP-13 and ADAMTS-5 in chondrocytes that had been exposed to IL-1*β* [49]. EXOs also attenuated the downregulation of COL2A1 and ACAN and the inhibition of proliferation and migration of chondrocytes. In addition, EVs enriched from citrate-anticoagulated PRP preparations were able to prevent the IL-1*β*-triggered morphological changes of OA chondrocytes, but this was not observed when using EVs from hyperacute serum [50]. In fact, citrate-anticoagulated PRP EVs were able to rescue the chondrocytes from the morphological phenotype that would be indicative of cell death. The protein expression of nuclear factor-κB decreased in the presence of EVs from both blood-products. These results have clear translational potential, as IL-1*β* is also one of the main mediators of cartilage destruction in OA patients [51]. 

While current results have mostly been obtained from in vitro models of acute cartilage inflammation or animal experiments with induced trauma, some data are emerging from experiments attempting to model chronic inflammation, which would be more relevant regarding advanced OA. When using the IL-1*β*-induced inflammation model of FLSs, Ragni et al. [33] were able to trigger decreases in the expression of pro-inflammatory factors IL-6, CCL-2, and CCL-5 after treating FLSs with adipose tissue-derived MSC EVs. In a more short-term study on OA chondrocytes treated with IL-1*β*, EVs derived from adipose tissue-MSCs were able to reduce the production of inflammatory mediators, including TNF-*α*, IL-6, PGE_2_, and nitric oxide, as well as to downregulate COX-2, mPGES-1, and inducible nitric oxide synthase [52]. Furthermore, there was a decrease in the total MMP activity and MMP-13 expression, and an increase in COL2 protein expression. While not comparable to the slow development of human OA, the potential of EV therapies in anti-inflammatory pain management seems significant.

EVs derived from the infrapatellar fat pad (IFP) of the knee joint have also been suggested as candidates for OA therapies, and the general role of the IFP in OA is being actively investigated. In 2011, it was documented how IFP-derived MSCs were able to alleviate OA in rabbits [53]. After surgically-induced OA (anterior cruciate ligament transection), rabbits were administered with intra-articular MSCs, and after 20 weeks they showed lower degrees of cartilage destruction, osteophytes, and subchondral bone sclerosis than control animals. At the time of the publication, the effects were not attributed to EVs, but when we examine later research, it is plausible that EVs could have been among the actual therapeutic agents in these earlier studies, as well. It was later established, for instance, that IFP MSC-derived EXOs were able to protect articular cartilage from damage and to ameliorate gait abnormalities in mice with surgically-induced OA by maintaining cartilage homeostasis, and this was suggested to be related to miR-100-5p-regulated inhibition of mTOR-autophagy pathway [54].

### 5.3. Cartilage Regeneration and Repair by EVs

During OA progression, articular cartilage experiences thinning and defects eventually appear with exposed bone causing more and more severe symptoms and dysfunction [1]. Repairing cartilage damage at this advanced stage of OA is a major challenge but initial results of EV treatment, once again, show significant promise. However, it must be emphasized that the results principally derive from experimental, acutely-caused traumatic cartilage lesions rather than from models of chronic disease that would be the case of practically all OA patients. When using MSC-derived EXOs on surgically-induced osteochondral defects in rabbits, EXOs in combination with HA produced significantly better results than HA alone and were able to induce sustained and functional cartilage repair after 12 weeks [55]. In a sodium iodoacetate rat OA model, similar beneficial effects were obtained after intra-articular BMSC EXO injections [49]. While the iodoacetate group showed osteophytes and a rough, ulcerated articular surface after 6 weeks, the joint injuries of the EXO-treated rats were repaired to some extent and they had a lower arthritis score. Repair of damaged cartilage was also observed in the study by Zhang et al. [56], where osteochondral defects were surgically induced on the femoral side of the rat knee joint. Thorough evaluation at 12 weeks after intra-articular human embryonic MSC EXO injections revealed in most cases complete restoration of cartilage and subchondral bone, with good surface regularity and structural integration of hyaline cartilage. Once again, the results were obtained after fresh trauma and they may not be directly applicable to the slowly progressing cartilage defects of OA. However, the relevance and future potential in post-traumatic OA are significant, as with chondrogenic progenitor cell-derived EVs from so-called superhealer mice, post-traumatic OA could be effectively attenuated in the surgical destabilization of the medial meniscus model [57]. In addition to cartilage, EVs are being studied for their potential to promote healing of tendons and ligaments, the degeneration of which can accompany OA development [5].

In fresh trauma, the damaged cartilage can, thus, be repaired in animal models with the use of intra-articular EVs. If this is to be applied to human medicine, the first stage for experimentation would probably not be advanced primary OA but fresh cartilage lesions that would eventually develop into secondary OA. End-stage primary OA with osteophytes, sclerotic bone, and the missing cartilage lining would probably present many additional challenges for cartilage replacement with EV therapies, but the gradual progression from fresh lesions to symptomatic OA with significant cartilage destruction will undoubtedly be investigated in the future. Based on recent information, there are several ongoing clinical trials on EV therapies [58], but these are not OA-related. In the ClinicalTrials.gov database (on 14 December 2020), there were 4004 ongoing clinical studies on the subject of “osteoarthritis” but very few if any with “exosome(s)”, “microvesicle(s)”, or “extracellular vesicle(s)” included. Yet 149 studies included mesenchymal or other stem cells as OA treatment options, and it is expected that EVs will also soon enter at least phase 1–2 clinical trials. Obviously, the road from clinical trials to actual routine treatments remains a long and tortuous one, but the increasing interest and the often astonishingly promising results from in vitro and animal studies indicate that actual EV therapies could become available within a few decades.

### 5.4. EVs in Targeted Drug Delivery and Potential Caveats of EV Therapies

In addition to the direct functions of EVs as therapeutic agents with anti-inflammatory or even regenerative properties, EVs have potential applications as transport vehicles of molecules, such as nanomedicine, to diseased sites in the body [59]. EV membrane peptides can be modified to recognize specific cell surface receptors to enhance their distribution to preferred destinations in the body [60]. In this respect, EVs could protect their medicinal cargo and guide it to reach the site of desired action. The production of these types of EVs is under investigation with techniques for which there is a strive for standardization [61]. The cellular uptake (internalization) of EVs would be the next step, and it remains undetermined, if this would always be necessary for the effects of EVs to take place on target cells [62]. For RNA to take effect, internalization would be necessary, but the same is not automatically true when it comes to EV-delivered medicines that could exert their effects via, e.g., receptor interactions. However, it is known that EVs can fuse with the plasma membrane or be internalized by endocytosis [62]. At present, potential treatments with EV therapeutic cargo have been mainly targeted at cancers [60], but the applications can be expected to increase in the future. With the focus on joint diseases, of special interest are SPMs and their precursors that have been isolated from EVs [63,64]. Norling et al. [63] constructed nanoparticles containing aspirin-triggered resolvin D1 or lipoxin A_4_ analog, which were able to dampen the inflammatory response in the mouse peritonitis and temporomandibular joint inflammation models. Generally, EVs from inflammatory exudates can contain SPM precursors with elevated levels during the resolution of inflammation. Thus, it can be suggested that administering nano-pro-resolving medicine to inflamed tissues, such as the OA joint, could provide pain relief and alleviate the pathological processes.

If EV products are to be utilized as therapeutic tools in the future, several aspects of safety and quality have to be taken into consideration. Like cell cultures and, for instance, blood-derived products, EVs can vary in composition and quality and there is the risk of contamination. However, in contrast to live cellular products, EVs are unlikely to transfer whole genomes or intracellular organisms to patients, their contents can be more easily manipulated, and their purification is more feasible than that of whole cells. Still, it will be necessary to establish standards for isolation, quality control, and storage of EVs with appropriate infrastructure—issues that are still a long way off [59]. To produce EVs with good manufacturing practice, both the methods of cell expansion and EV isolation would have to be carefully documented, including culture media, substrates, and dissociation enzymes on one hand; and the process of EV isolation and purification steps on the other [58]. Useful products even with evidence-based beneficial effects will not become available without these aspects of drug safety and delivery being in place. Similarly, the actual therapeutic product should be meticulously characterized. Another issue is to assess, how the emerging EV-based therapies supplement existing ones, and if the novel diagnostic and prognostic tools are better and more practical than the existing tools of symptomatology and imaging techniques. Obviously, for advanced OA, the existing diagnostic tools seem to be quite adequate, but early-stage OA with minimal radiographic changes could potentially benefit from novel diagnostic measures allowing interventions to prevent the development of cartilage destruction. 

To conclude, the EV option shows beneficial potential regarding OA pain management. It may obviously require EV administration in a relatively invasive manner compared to peroral NSAIDs, but that is also the case of presently available intra-articular steroid or GAG products. The promise of actual cartilage repair is even greater with different MSC EVs being able to enhance cartilage restoration, albeit this has principally been demonstrated in small-scale animal experiments after acute surgically- or chemically-induced cartilage defects. The acquisition of EVs, as discussed above, is another issue that should be assessed. Not only is the isolation procedurally cumbersome, but biologically active material could also provide risks of contamination and disease transmission—albeit probably smaller than in the case of whole cells—that need to be controlled before these treatment options can be widely applied to the population.

## 6. Perspective

EV research is emerging as a significant contributor to our understanding of OA pathogenesis, its progress, and treatment. When considering the future applications of EVs in translational research and therapeutic interventions of OA, it is important to assess them against the existing methods of diagnostics and therapies. What current medical practice is missing are early-stage diagnostics and biomarkers in the asymptomatic or relatively symptom-free stages, and here especially circulating EV profiles could be of use in the future. In contrast, advanced OA can be easily diagnosed based on patient symptomatology and various imaging techniques, and it is questionable if EVs would have practical and economical applications here.

Regarding therapies, the current treatment is at best symptomatic and able to decelerate the progress of OA. Pain management is relatively effective with physiotherapies and NSAIDs, but medical interventions have adverse effects, for instance, in the gastrointestinal system, and intra-articular injections are either insufficiently justified or can cause side effects (corticosteroids). The two stages of OA, the asymptomatic and the one characterized with increasing pain, would benefit from the anti-inflammatory potential of EVs, perhaps initially as supplements to existing therapies.

The final stage of OA, that of cartilage destruction and sclerotic bone changes, is the one that would be the principal target of EVs. First, if it would become feasible to prevent cartilage destruction in the first place and to shift the balance to regeneration in the early stages, surgical interventions would become obsolete. Second, the possibility of EVs triggering the formation of new cartilage is the most attractive. At the moment, existing data are still in early stages and mostly derived from cell cultures that are inadequate models for the chronic inflammation encountered in OA. Still, it can be argued that since the advent of arthroplastic surgery, no novel treatments have shown as much promise and research potential as EV-based therapies to treat OA.

## Data Availability

No new data were created or analyzed in this study. Data sharing is not applicable to this article.

## Abbreviations

ACAN	Aggrecan
ACV	Articular cartilage vesicle
ADAMTS	A disintegrin and metalloproteinase with thrombospondin motifs
BMSC	Bone marrow mesenchymal stem cell
CCL	Chemokine (C-C motif) ligand
COL2A1	Collagen 2A1
COX-2	Cyclooxygenase-2
ECM	Extracellular matrix
EV	Extracellular vesicle
EXO	Exosome
FLS	Fibroblast-like synoviocyte
GAG	Glycosaminoglycan
HA	Hyaluronic acid
IFP	Infrapatellar fat pad
IL	Interleukin
lncRNA	Long non-coding RNA
MCP	Monocyte chemoattractant protein
miRNA	MicroRNA
MMP	Matrix metalloproteinase
mPGES-1	Microsomal prostaglandin E synthase-1
MSC	Mesenchymal stem cell
NSAID	Non-steroidal anti-inflammatory drug
OA	Osteoarthritis
PG	Proteoglycan
PGE_2_	Prostaglandin E_2_
PRP	Platelet-rich plasma
PUFA	Polyunsaturated fatty acid
RA	Rheumatoid arthritis
SF	Synovial fluid
sGAG	Sulfated glycosaminoglycan
SOX9	SRY-related HMG box 9
SPM	Specialized pro-resolving mediator
TGF-*β*	Transforming growth factor *β*
TNF-*α*	Tumor necrosis factor *α*
